# Exploration of emotion regulation experiences associated with borderline personality features in a non-clinical sample

**DOI:** 10.1186/s40479-016-0040-6

**Published:** 2016-08-15

**Authors:** Carly M. Porter, Carol A. Ireland, Kathryn J. Gardner, Mike Eslea

**Affiliations:** 1 School of Psychology, University of Central Lancashire, Preston, UK; 2 Ashworth Research Centre, Mersey Care NHS Trust, School of Psychology, University of Central Lancashire, Preston, UK; 3 CCATS: Coastal Child and Adult Therapeutic Services, Preston, UK; 4 ClinPsyD, The University of Manchester, Second Floor Zochonis Building, Brunswick Street, Manchester, M13 9PL UK

**Keywords:** Emotional regulation, Borderline features, Emotions, Attention

## Abstract

**Background:**

Emotion dysregulation is a core feature associated with borderline personality features (BPF). Little research has explored how individuals with high levels of BPF regulate their emotions. This study aimed to explore how individuals with high versus low levels of BPF compare on the strategies they use to regulate emotions and in their experiences of emotion regulation.

**Methods:**

Twenty-nine university students were recruited and assessed for the presence of BPF using self-report questionnaires. Each participant took part in a semi-structured interview about their experiences of emotion regulation. All interview transcripts then underwent thematic analysis. In addition chi square analyses were conducted to explore the association between level of BPF (High vs Low) and each qualitative theme identified.

**Results:**

Findings indicated similarities in the types of emotion regulation strategies used by the high and low-BPF groups. However, the groups differed in their experiences and thought processes surrounding emotion regulation. High-BPF participants were found to describe a need to communicate negative emotions with others and demonstrated difficulty maintaining attention on positive experiences. In addition there was a trend towards High-BPF participants demonstrating less forward-planning in emotion regulation.

**Conclusions:**

This study provides insights into some of the unique aspects of emotion regulation in individuals with high BPF that may make emotion regulation attempts less successful.

## Background

Borderline personality disorder (BPD) is a severe psychiatric disorder involving impulsive behaviour and instability in interpersonal relationships, identity and emotions [2]. A seminal theory of the development and maintenance of BPD is biosocial theory [30]. This theory states that borderline personality features (BPF), which collectively form the BPD diagnosis, emerge from and are maintained by the primary problem of emotion dysregulation. It is theorized that for these individuals emotion dysregulation occurs as a result of biologically determined emotional vulnerabilities that increase the demand for effective emotion regulation and an invalidating environment during childhood that fails to teach the individual how to regulate emotion effectively. As a result, emotion regulation demands are not met.

It has been found that emotion regulation problems are highly associated with the presence of BPF [11]. In addition, dialectical behaviour therapy (DBT), which is based on biosocial theory and teaches emotion modulation skills, has been found to be successful in reducing para-suicidal behaviour, experiences of anger and days spent in psychiatric inpatient facilities and improving social adjustment [8, 31, 32, 49, 51]. This suggests that supporting individuals to regulate their emotions leads to reductions in several problematic BPF. However, the exact mechanisms of change within DBT remain unclear [33]. Therefore it is unclear which specific aspects of emotion regulation are problematic within BPF.

In nonclinical samples the type of emotion regulation strategy used has been found to influence emotional experience and interpersonal functioning [24]. For example, the use of reappraisal has been associated with increased positive emotion experiences, decreased negative emotion experiences and improvements in interpersonal functioning. Conversely, the use of suppression has been associated with less positive emotion, more negative emotion and poorer interpersonal functioning [24]. This has been replicated in more recent research, which reports that increased use of suppression is associated with weaker interpersonal relationships and reappraisal with stronger interpersonal relationships (English, John, Srivastave, & Gross, 2012). Consistent with biosocial theory [30], this suggests that how an individual attempts to regulate their emotions, or more specifically the types of strategies that they use, is associated with emotional experience and interpersonal functioning. These are two central parts of the borderline construct. As a result strategy selection was considered as one area of emotion regulation that may be problematic for individuals with high levels of BPF.

In line with this, past research has reported that individuals with high levels of BPF have limited access to functional emotion regulation strategies [16, 20, 40]. Further, the presence of BPF has been associated with the use of a wide range of dysfunctional emotion regulation strategies, such as rumination [3, 41, 43], experiential avoidance [9, 26], thought suppression [12, 38] and deliberate self-injury [6, 36]. However, each of these studies looks at specific *dysfunctional* strategies in isolation, thereby ignoring the potential for a range of both dysfunctional and functional emotion regulation strategies to be used. It has recently been identified that emotion regulation strategies are rarely used in isolation, highlighting the need to investigate the full range strategies used by an individual [1]. If these individuals are also using functional strategies, this may be one area that can be capitalised on during treatment.

A limited amount of research has investigated the range of strategies used by individuals with high levels of BPF or BPD. One such study used reports made by experienced clinicians to compare the use of five types of emotion regulation strategies used by individuals with a diagnosis of BPD to those used by individuals with dysphoric disorder [13]. It was reported that individuals with a diagnosis of BPD use dysfunctional strategies to a greater extent and functional strategies to a lesser extent than those with a diagnosis of dysphoric disorder. However there are methodological limitations that need to be considered. The study relied solely on clinicians rating of observed emotion regulation strategies and did not consider the perspective of the individual. Further to this the clinicians were not blind to the diagnosis of the patients they were rating and as such it is possible that these ratings may be biased. In addition four out of the five types of emotion regulation strategies investigated were considered dysfunctional, leading to a potentially bias view of the range of strategies used in this population. Despite its limitations, the empirical evidence presented above suggests that individuals with high levels of BPF, which may or may not be sufficient for diagnosis, use a limited number of dysfunctional emotion regulation strategies.

However, research has also suggested that individuals with high levels of BPF demonstrate sufficient knowledge of emotion regulation strategies. Beblo et al. [4] reported that individuals with a diagnosis of BPD were found to be comparable to healthy controls in their ability to select functional emotion regulation strategies, in a flexible manner according situational demands presented in vignettes. This was despite reporting high levels of emotion regulation difficulty on self-report measures. Further to this, research has found that when instructed to use specific emotion regulation strategies over a set period of time, individuals with high levels of BPF are able to use a range of strategies to successfully regulate positive and negative emotions [10, 27]. This appears inconsistent with the aforementioned research, which suggested that these individuals use dysfunctional emotion regulation strategies.

However, the quantitative research methods used to date to explore strategy use in this population may not provide an accurate profile of strategy use for several reasons. Firstly, a limited number of strategies were explored in these studies. Secondly, of the strategies explored there is a heavy focus on dysfunctional strategies at the expense of functional strategies. Thirdly, there has been a major focus on the regulation of negative emotions, largely ignoring the process of positive emotion regulation despite its importance for psychological wellbeing [7, 17]. Finally, research to date has not explored how individuals with high levels of BPF experience emotion regulation. This may be beneficial to understand why some strategies are favoured over others.

The present study adopts a qualitative approach using semi-structured interviews to enquire about how individuals with high versus low-levels of BPF compare on the types of strategies they use and in their experiences of positive and negative emotion regulation. This approach extends past research by allowing individuals to talk openly about how they regulate their emotions from their own perspective and in their own words; this is crucial to achieve depth of understanding of the full range of strategies used by these individuals and why they used them.

## Method

### Participants

A sample of 29 (*n* = 16 female, *n* = 13 male) participants were recruited from a university student population in the northwest of England. The sample ranged from age 18 to 46 (*M* = 24.62; *SD* = 6.90). All participants were enrolled on higher education courses. Regarding Ethnicity 76.7 % regarded themselves as white British, 13.3 % as white other, 3.3 % as Chinese, and 3.3 % as Black British. One participant failed to provide ethnic information. Regarding Academic status 3.3 % were enrolled on a foundation degree level course, 76.7 % were enrolled on an undergraduate BSC/BA degree, 6.7 % postgraduate Masters Level degree, 6.7 % were enrolled on a Postgraduate Doctoral Level degree, 3.3 % other. Only 23.3 % of participants were studying Psychology with the remaining sample demonstrating a range of disciplines across the university.

### Materials

#### Interview topic guide

Following a review of the literature key areas for exploration were identified; emotional experience, strategies employed for emotion regulation and personal experience of the emotion regulation process. A topic guide was developed to direct conversation toward these areas of interest. The topic guide included four lead questions each followed by a series of possible prompts used flexibly to promote further discussion. The first two questions were more general and were designed to ease participants into the interview and encourage them to focus their thoughts on the topic of emotion regulation. The latter two questions were more specific, encouraging participants to describe their own emotion regulation behaviours and experience.

1. ‘*What does the term emotion mean to you?’* Prompts focused on encouraging individuals to think about different types of emotions, and to compare emotions to encourage more detailed information on their emotional experiences.
2. *‘Do you think humans have the ability to influence their emotions?’* This question was used to encourage participants to think about how emotions might be influenced. Prompts encouraged discussion about the individual’s perceived control over emotions.
3. *‘Can you tell me about a time when you have tried to influence your emotions?*’ This question was designed to encourage discussion about strategies used by the individual to regulate their own emotions. Prompts following this question encourage participants to consider positive and negative emotions and the specific actions they take to influence emotion. Where possible participants were encouraged to provide examples of situations strategies used.
4. *‘Did that work for you/how do you know that that strategy was effective?’* This encouraged discussion of the perceived effectiveness of strategies and reasons for their use. It also provided an opportunity for difficulties in emotion regulation to be discussed.

### Self-report measures

Self-report questionnaires were used to assess the presence of BPF. Because self-report assessments have been found to produce a high rate of false positives [15], two measures of BPF were used and their scores aggregated; a method that has been found to reduce false positives and improve reliability [39]. This meant that individuals needed to score above the cut-off score on both measures in order to be included in the high BPF group. Participants scoring below the cut off on one or both of the self-report measures were considered to demonstrate low-level BPF [35]. The two self-report measures used to differentiate between high and low BPF in this study were the Personality Assessment Inventory-Borderline Scales (*PAI-BOR;* [34]*) and the Personality Diagnostic Questionnaire – 4 Borderline Scale (PDQ-BS;* [25])*.*

The 24-item PAI-BOR scale was taken from the larger 344 item personality assessment inventory; designed to assess personality pathology according to DSM-IV diagnostic criteria [34]. The PAI-BOR scale was used to provide a global score of BPF. Each item is a statement, e.g. ‘My mood can shift quite suddenly’, to which participants respond using a four-point Likert scale (0–3) to illustrate how much the statement was true of them (0-False to 3–Very True). The Global score of BPF has good internal consistency (α = .86) and test re-test reliability (*r* = .82) in a non-clinical sample [34]. Consistent with past research internal consistency within the current sample was found to be good (α = .84). PAI-BOR scores >38 were found to indicate the presence of BPF [47] and were used in this study.

The PDQ-BS was taken from the larger personality diagnostic questionnaire- fourth edition [25]; a 99-item self-report screening measure based on criteria for personality disorder according to the DSM-IV [2]. The PDQ-BS contains 9-items designed to screen for the presence of BPF. Each item is a statement e.g. ‘I’ll go to extremes to prevent those who I love from ever leaving me’. Considering each statement in the context of the ‘past several years’ participants are required to indicate whether the statement is ‘True’ (1) or ‘False’ (0) of them. Previous research using the PDQ-BS as a screening tool in non-clinical populations has reported good internal consistency (α = .81) [19], in the current study internal consistency was found to be lower (α = .54). A score >5 indicates clinically significant levels of BPF [28] and was therefore used in this study. In addition subscales for other cluster B personality features were also included to allow exploration of the specificity of study findings to BPF.

#### Positive and negative affect scales (PANAS; [50])

The PANAS consist of two 10-item subscales: positive affect and negative affect. The scales have been reported to demonstrate good internal consistency for the current moment in a non-clinical population (α = .89, .85, respectively) [50]. The PANAS was used to assess current affective state. The positive and negative affect scales demonstrated adequate internal consistency in the current sample (α = .77, α = .77, respectively).

### Procedure

Ethical approval was obtained from the University ethics committee. Participants were recruited from the student population at the university. In order to maximise the number of participants likely to demonstrate high levels of BPF a targeted recruitment approach was adopted. This approach involved developing recruitment advertisements, which included questions relating to BPF (e.g. do you experience intense emotions?) These advertisements were displayed across the university campus and sent out via a global email system. All participants responding to recruitment advertisements were provided with a detailed information sheet and given the opportunity to ask any questions. Following this all participants who volunteered to take part were included in the study sample. On arrival participants were taken to a quiet interview room and asked to complete the PANAS self-report questionnaire to assess current affect at the time of the interview. Participants then took part in a one-to-one semi-structured interview lasting approximately 30–45 min, which was audio recorded to allow transcription and analysis at a later date. Finally participants were asked to complete a second questionnaire which included the personality assessments.

### Qualitative methodology

Qualitative methodology was used in this study to allow a rich description of emotion regulation techniques to be obtained from the perspective of the individual. This facilitates understanding of thought processes that may underlie decisions about emotion regulation as well as understanding of individual experiences of emotion regulation. All interviews were conducted, recorded and transcribed verbatim by the first author, who was blind to BPF classification at the time of transcription. Thematic analysis was then used to identify key units of meaning.

Thematic analysis is an analytical procedure whereby through careful reading and re-reading of the data key topics or themes are identified as being important to the description of a given subject [14]. These themes then become categories for further analysis. Therefore thematic analysis is well placed to explore the use of emotion regulation strategies and associated experiences, which may then be compared across groups. Thematic analysis was conducted based on procedures outlined by Braun and Clarke [5]. Familiarisation with the data were achieved through transcription of all interview recordings by the primary researcher and listening to interview recordings twice, prior to formal coding. Consistent with the guidelines presented by Braun and Clarke [5] and to reduce bias toward existing theoretical perspectives no initial coding scheme was proposed. Instead, using an inductive approach, initial codes were developed sentence-by-sentence. This meant that the initial codes developed were literal descriptions of the information contained in that particular sentence (e.g. distracts self from unwanted thoughts). This ensured that the codes reflected the data as accurately as possible and that nothing was missed which may later develop into a theme. The initial codes were then collated across transcripts by grouping data with similar codes together to identify overall themes within the data. These themes were then reviewed in the context of the larger dataset to ensure that they were accurate representations. The coding process and identification of themes was conducted blind to BPF classification, i.e. whether the transcripts belonged to the high or low BPF group, to avoid bias in coding. To ensure scientific rigour 10 % of the transcripts were also coded by another researcher, who was also blind to the BPF classification. The second researcher had prior experience in this type of qualitative analysis but was not considered to be an expert in emotion regulation and BPF. This use of a theoretically neutral second coder was considered important to identify any potential theoretical bias in the codes assigned by the primary researcher, given the inductive perspective adopted. In addition codes made by the primary researcher were not made available to the second coder to reduce the potential for bias. In accordance with the recommendations made by Frommer and Rennie [18] any disagreements on the codings between the two researchers were considered and discussed until agreement was reached [18].

Although the primary aim of this study was to explore individual’s experiences of emotion regulation using a qualitative approach. The statistical significance of findings was also tentatively explored. Namely chi square analyses were used to explore whether or not there was a statistically significant association (*p* < .05) between themes identified and group membership.

## Results

### Sample groupings

For the purpose of this study participants scoring >5 on the personality diagnostic questionnaire and > 38 on the personality assessment inventory were classified as high BPF. Therefore individuals scoring below the cut-off on one or both measures were classified as low BPF. Six participants were found to score above the cut off on only one measure and thus were included in the low BPF group; one of these participants scored above the cut off for the PDQ only and five scored above the cut-off on the PAI only. This led to *n* = 16 participants in the low BPF group (*n* = 8 Male, *n* = 8 Female) and *n* = 13 in the high BPF group (*n* = 5 Male, *n* = 8 female). There were no significant differences in gender across BPF grouping (*χ*^2^ (1) = .39, *p* = 534). An independent *t*-test revealed that the high BPF Group scored significantly higher than the Low BPF group on PDQ_BOR (*t*(27) = .25, *p* = 806) and the PAI_BOR (*t*(27) = 5.17, *p* < .01). Means and standard deviation of age, current affect, and cluster B personality scores were computed for each group to ensure homogeneity of these variables across groups (Table 1). The group means indicate that for age (*t*(27) = 1.09, *p* = .285), Current Positive affect (*t*(27) = .151, *p* = .881), current negative affect (*t*(27) = 1.51, *p* = .144), and cluster B personality scores (PDQ_NAR (*t*(27) = .59, *p* = .558); PDQ_AS (*t*(27) = .25, *p* = .806); PDQ_HIS (t(27) = 1.95, *p* = .062)) did not differ significantly between the two groups.

**Table 1:** Means and standard deviations by BPF classification for age, current affect, and borderline personality scores

	Low BPF group		High BPF group		t-tests to compare groups
	Mean	S.D	Mean	S.D	
Age	25.88	6.69	23.08	7.10	*t*(27) = 1.09, *p* = .285
Positive affect	30.25	6.69	29.92	4.46	*t*(27) = .151, *p*=. 881
Negative affect	14.31	3.46	16.77	5.29	*t*(27) = 1.51, *p* = .144
PDQ-AS^a^	2.31	1.85	2.46	1.39	*t*(27) = .25, *p* = .806
PDQ-HS^a^	3.31	1.85	2.15	1.28	*t*(27) = 1.95, *p* = .062
PDQ-NS^a^	2.31	1.92	1.61	1.04	*t*(27) = .59, *p* = .558
PDQ-BOR^a^	3.31	1.19	6.46	1.33	*t*(27) = 6.71, *p* < .01
PAI-BOR^b^	34.40	6.91	48.62	7.84	*t*(27) = 5.17, *p* = < .01

*Note.* ^a^ *PDQ* personality diagnostic questionnaire, *AS* anti-social scale, *HS* histrionic scale, *NS* narcissistic scale, *BOR* borderline scale. ^b^ *PAI-BOR* personality assessment inventory borderline scale

### Thematic analysis of transcripts

Several themes emerged from the dataset, some of which were beyond the scope of the research questions. Therefore only themes relating to the topic of emotion regulation underwent further analysis. In order to explore similarities and differences in the experiences of individuals with high versus low levels of BPF, the evidence in each theme was split according to BPF classification. Analysis continued to identify similarities and differences between the two classifications. At this stage analysis progressed from description of the data to interpretation, where an attempt was made to theorise the significance of the differences and their broader meanings [37]. This led to the development of new themes. Emerging themes were continually reviewed in the context of the full transcripts in a recursive process. This ensured that findings were accurate representations of the full data set.

There were four themes identified in the dataset. These were: type of emotion regulation strategy, immediate vs. long term emotion regulation, difficulty maintaining positive focus and communication of negative emotion. Each of these themes are discussed in turn, highlighting similarities and differences between high and low BPF individuals within each theme.

#### Theme I: Type of emotion regulation strategies

Each transcript provided descriptions of different ways in which the individuals attempt to alter their emotions. Across all transcripts the primary purpose was to maximise the experience of positive emotions and minimise negative emotions, for example, *‘I think everybody should really aim towards being happy because to live a good life you have got to be happy. You know if you are unhappy with your life it’s, err, it’s hard to deal with’ (P25)*^1^*.* Strategies reported for the regulation of positive and negative emotions were analysed separately. Across the full dataset four types of strategy were identified for the regulation of positive emotion and seven types of strategy were identified for negative emotion regulation. Definitions of each strategy and supporting evidence for positive and negative emotion regulation can be found in Tables 2 and 3 respectively. When looking at strategies for positive emotion regulation (Table 2), it can be seen that evidence for each type of strategy came from both the high and low BPF group. However, a higher proportion of individuals from the low BPF group provided evidence of using each of the strategies identified. When looking at strategies for negative emotion regulation (Table 3), it can be seen that there are roughly equal percentages of the high and low BPF participant describing each strategy except for: 1) Problem solving, for which a relatively smaller proportion of the high BPF group describe using and 2) Suppression, for which a relatively higher proportion of the high BPF group describe using. Chi Square analyses revealed no association between BPF grouping and reporting of any strategy (all p’s > .05)

**Table 2:** Strategies identified for the regulation of positive emotion across all transcripts

Strategy	Operational definition	Example	Evidence source^a^
Situation selection	Choosing or changing a situation to initiate or maintain a positive emotion.	*‘I try to fill my time with things that make me happy.’ (P14)*	H = 61.52 %,L = 68.75 %
Directed attention	Choosing to focus attention in order to embrace current past or future positive emotion.	*‘I sometimes tell myself how happy I am and how lucky I am to have the things I have so I kinda like it makes me more happy’ (P8)*	H = 38.46 %, L = 56.25 %
Substance use	The use of substances (e.g. food, caffeine, alcohol, cannabis) to initiate or enhance positive emotion.	*‘…like playing games in particular is enhanced by smoking weed because it makes you worse and that makes it funnier’ (P14)*	H = 7.69 %, L = 18.75 %
Passive	No explicit action taken to alter positive emotion.	*‘yeah I don’t know happiness I have never I don’t really seem to think about happy as much as I do try to get rid of negative’ (P20)*	H = 30.77 %, L = 37.50 %

*Note*:^a^Indicates the number of high (H) out of *n* = 13 and low (L) out of *n* = 16 BPF transcripts that provide evidence for each strategy

**Table 3:** Strategies identified for the regulation of negative emotion across all transcripts

ER strategy	Definition	Example	Evidence source^a^
Problem solve	Taking action to attempt to alter an emotion eliciting situation in order to change its emotional impact.	*‘The last few years of my marriage I was sad that the situation had got where it had…..I tried to make recompense and save the marriage…’ (P30)*	H = 46.15 %, L = 62.50 %
Avoidance	The deliberate attempt to avoid an emotion or situation causing emotion by removal of self, deployment of attention, or emotion escape via substance use.	*‘If I get like really angry like for example in a dispute or argument with a certain person I will prefer to just walk away…’ (P15)*	H = 100 %, L = 100 %
Emotional Expression	The deliberate outward projection of emotion via emotional behaviour, physical exercise or verbal communication.	*‘You feel like the need to express it so for me I for me I would say something to the person that had frustrated me’ (P18)*	H = 84.62, L = 87.50 %
Emotion Suppression	The effortful action of hiding behavioural displays of internal emotional states from others.	*‘I tried to hide it [sadness]. Just I dunno I was trying to be strong’ (P20)*	H = 46.15 %, L = 37.50 %
Rumination	A persistent focus on past negative situations and negative aspects of self and on their possible causes and negative consequences.	*‘I just get angry and I don’t know, just keep thinking about it again and again, it’s kinda like trying to find a solution to a possible problem’ (P8)*	H = 46.15 %, L = 50 %
Reappraisal	Deliberately changing how one thinks about an emotional situation (in the presence of emotion) to alter its emotional impact.	*‘I did look on the bright side of it, to know that they weren’t in pain any more, to know that they weren’t hurting. So I was thankful for that’(P9)*	H = 92.31 %, L = 93.75 %
passive/helpless	The awareness of an emotion whilst making no conscious effort to influence it.	*‘I don’t tend to [regulate negative emotions] I just wait until something better comes along’ (P19)*	H = 46.15 %, L = 50 %

*Note:* ^a^Indicates the % of transcripts from the high (H) and low (L) BPF groups that provide evidence for each strategy

#### Theme II: Immediate vs. long term emotion regulation

Participants from both the high (38 %) and low (50 %) groups (*χ*^2^(1) = 3.86, *p* = .534) described using techniques that led to immediate changes in unwanted emotion, despite showing awareness that these strategies may have dysfunctional long term consequences, for example, *‘I do, I know I shouldn’t do this one but I do just go out and spend a lot of money and that changes the way I feel. Like say if I am really mad about something I will just bugger off and buy loads of weird things and then come home with them and then I don’t even know why I have got them. But that is good for me because I do feel happy once I have done it. I come home and I do feel very happy (laughs) but then I am like why have I just spent all of my money, what am I going to do now?’ (P23)*.

However, 44 % of the low BPF participants versus 15 % of high BPF participants (*χ*^2^(1)2.70, *p* = .101) also demonstrated consideration of how to make changes to emotion long term, for example *‘so… I will think how can I cure that emotion? How can I cure that emotion? How can I stop it- it’s like well what can I do to make sure that doesn’t happen again’ (P36).* This long term consideration was absent in transcripts from most high BPF participants.

#### Theme III: Difficulty maintaining positive focus

Some participants, particularly in the high BPF group (62 % of the high versus12% of the low BPF participants (*χ*^2^(1) = 7.64, *p* = .006)) demonstrated difficulties with maintaining attention on positive events and experiences. In descriptions of positive emotion regulation this was evidenced by descriptions of problems maintaining focus on the current positive event; instead being distracted by unrelated negative situations, for example, *‘Yes when I am in situation where I feel like the happiest person in the world I still have those negative thoughts that should be at the back of your mind but they are at the forefront and I am constantly just telling myself just take it all in at this moment in time so that you can think back on it later and remind yourself that this is what happiness feels like.’(P26).* Alternatively some high BPF participants demonstrate a focus on negative elements within the positive situation, for example, *‘I think a lot of the time when I do [try to increase positive emotions], it doesn’t really work. I think if I plan something and think oh this will be great and y’know plan a day and think I’m going to really enjoy this- already it’s like not going to meet that expectation so I find that quite hard. I mean obviously I do plan things that I hope I will enjoy.’ (P11).* Here the focus is on where the positive experience has fallen short of expectations rather than the positive experience itself.

Difficulty in maintaining a focus on positive experiences and events was also demonstrated in high BPF descriptions of negative emotion regulation. This impacted on reappraisal abilities with reappraisal attempts turning into rumination, for example, *‘No, so for quite a long time I really, really, really resented my parents for doing that. I mean I went to university and I went abroad and I met a lot of people that are very special in my life and you realise that if things were different then you wouldn’t have been meeting these people….. and it sort of allowed me to let go of the resentment for my parents. But you know that you don’t want things to have changed, but on another level you are so aware, you are very aware that your parents are the type of people that did this, and that you can’t turn to them for support and that you have to be independent and that I am very much sort of on my own in this.’ (P14).*

#### Theme IV: Communication of negative feelings

Both high (77 %) and low (81 %) BPF participants (*χ*^2^(1) = 9.184, *p* = .002) described expressing negative emotion in order to gain an emotional release. For example a participant in the low group stated: *‘it’s just an offload isn’t it - it’s just offloading to someone you know or just, it’s just erm yeah you are just getting it out of your head aren’t you, the idea is that if you get it out of there and into there then it’s not in here anymore’ (P13) and a participant from the high group stated: ‘So to be able to do that would probably make me feel better I think, at least while I was doing it; it would be like a release of emotion.’ (P14).*

However, 46 % of the high BPF participants versus 8 % of low participants (*χ*^2^(1) = 6.0, *p* = .014) also describe emotion expression in order to communicate their negative emotion with others, for example, *‘that [throwing an object at another person] was just to stop her, just to make her see that I was suffering and to just y’know even any reaction.’(P14) and ‘I think when you get sort of a reaction a lot of the time like if somebody says something that annoys me or upsets me once I have got a reaction out of them even if the reaction is not nice to me I feel like I have achieved it and then I can move on from that emotion and think about something else.’(P11).* Here, the goal of the emotion expression is to get the emotion acknowledged by another individual. The respective percentages of transcripts from the high (H) and low (L) BPF groups that provide evidence for each strategy and Chi Square statistics are summarised in Table 4.

**Table 4:** Percentage of transcripts from the high (H) and low (L) BPF groups that provide evidence for themes II-IV

Theme (Subtheme)	Percentage of low BPF group coded under this theme (*N* = 16)	Percentage of high BPF group coded under this theme	Chi square to explore association between BPF group and theme
Theme II: Long term Emotion regulation focus	44 %	15 %	*χ* ^2^ (1) = 2.70, *p* = .101
Theme III: Difficulty maintaining positive focus	12 %	62 %	*χ* ^2^ (1) = .64, *p* = .006
Theme IV: Communication of negative emotions	46 %	8 %	*χ* ^2^ (1) = 6.00, *p* = .014

## Discussion

Little research has explored how individuals with high levels of BPF attempt to regulate their emotions during their everyday lives. This study used qualitative and quantitative methodology to explore if individuals with high versus low levels of BPF differ in the emotion regulation strategies they use and subsequent experiences of emotion regulation.

Findings indicate that there is little difference in the *types* of strategies reported by high and low BPF participants, with evidence for all strategies identified coming from both the high and low BPF groups. This supports previous research suggesting that individuals with a diagnosis of borderline personality disorder have sufficient knowledge of emotion regulation strategies despite reporting high levels of difficulty in regulating emotions [4]. The findings of the current study suggest that individuals with high levels of BPF use a range of strategies in everyday situations including those considered to be functional (e.g. reappraisal) and dysfunctional (e.g. suppression or rumination). This is in contrast to other research, which has predominantly focused on the use of dysfunctional strategies (e.g. [6, 13, 38]).

Despite similarities in the types of strategies described by high and low BPF participants, several differences were identified in the thought processes leading up to strategy use and in experiences of using the strategies. Whilst both groups of participants sought immediate change for unwanted emotions, there appeared to be a trend towards the low BPF group being more likely to discuss preventing unwanted emotional states in the future. This consideration of future emotional responses is illustrative of antecedent-focused emotion regulation (i.e., acting to influence an emotion before it is fully active), which has previously been reported as more effective [23]. It is possible that the lack of consideration for future unwanted emotion may result from low levels of distress tolerance which is characteristic of those with BPD [22]. This is because focusing attention on unwanted emotion may cause mild distress, and as a result individuals with high levels of BPF may be unwilling to experience this distress in order to achieve long term emotion regulation. Alternatively, these individuals may have a less understanding of their internal and external emotional triggers, possibly due to emotional invalidation in early childhood [30]. This would make it more difficult to predict when future emotion is likely to occur and thus would result in a reactive rather than proactive approach to emotion regulation, which is consistent with findings.

Findings from this study also demonstrate that many of the high BPF group reported difficulty regulating emotion due to problems with diverting attention away from negative, and towards positive emotional stimuli. This is consistent with past experimental research suggesting that individuals with a diagnosis of BPD demonstrate deficits in attentional control and inhibition of irrelevant aversive information [46]. Similarly, the emotional cascade model suggests that individuals with high levels of BPF experience difficulty in diverting their attention away from intense negative affect due to a tendency to ruminate [41, 42]. The difficulty in diverting attention away from negative and towards positive emotional stimuli identified in the current study may be considered illustrative of this hypothesised internal battle to divert attention away from ruminative thoughts.

Finally, transcripts revealed different thought processes underlying the use of emotion expression as a regulation strategy. When using emotion expression both high and low BPF participants describe behaving in a manner that facilitates the ‘release’ of emotion e.g. shouting, throwing objects. However, it was found that a number of high BPF participants described using these behaviours to communicate their negative emotion with others. This is consistent with past literature on self-injury, which suggests that individuals with a diagnosis of borderline personality disorder deliberately injure themselves to reduce negative emotional states, express emotion and to communicate distress with others [6, 29, 36]. This need for acknowledgement from others for internal emotional states may be rooted in the invalidation of internal emotional experiences during childhood [30], which may have prevented the learning of how to self-validate emotional states in later life; when this skill is lacking, the individual to seeks external validation for internal emotional states via extreme and maladaptive emotional expression. The use of emotion expression in this manner is likely to contribute to interpersonal problems, another key borderline personality feature, as it may involve extreme displays of negative emotion towards others.

The current study has limitations. Firstly, as is typical for qualitative studies, this study was conducted with a small sample size. As a result, the ability of these findings to be generalised is more limited and statistical analyses should be treated with caution, yet smaller samples of this nature allow for a richness of data. In addition, the use of non-clinical student sample limits immediate clinical utility of findings. However, the findings highlight specific problematic areas of emotion regulation, which appear to be associated with the presence of BPF, which can be targeted in future clinical studies where more severe levels of BPF are present. Yet the findings of this research may be more directly utilised in non-clinical support services, where individuals may present with high levels of BPF which do not necessarily meet diagnostic threshold, but may still be problematic in terms of increasing the interpersonal problems, risk of mood disturbance and having a negative impact on academic performance [48].

Secondly, the current study could not control for the presence of other cluster B personality disorder features and negative affect. Therefore it cannot be assumed from this sample that findings are specific to BPF. However, the presence of these features were assessed and found to be comparable across the high and low BPF groups. This was unexpected due to the higher levels of negative affect and co morbidity with other personality disorders previously reported in high BPF populations [21]. It is speculated that the comparable levels of negative affect across the high and low BPF groups may have resulted from the small sample of high BPF individuals scoring more highly on features such as impulsivity and poor self-identity rather than mood related features of BPD. Future research exploring emotion regulation in relation to BPF should consider its relation to individual criteria for BPF, by looking an individual subscale score e.g. impulsivity, as well as the construct as a whole using global BPF scores (as was done in this study). This would facilitate a more detailed understanding of how emotion regulation problems may act to maintain BPF, as suggested in biosocial theory [30]. For example, it may be the case that some problematic patterns of emotion regulation are associated with specific BPF rather than the construct as a whole.

Thirdly, the self-report nature of this dataset means that emotion regulation attempts described were explicit (conscious and effortful) attempts to alter emotional states. However, some of these attempts may have been conducted implicitly at the time and are brought into explicit awareness only on reflection. Finally, due to the qualitative nature of this research it was not possible to quantify emotional experiences described. This means that although high and low BPF participants may describe using the same strategies to regulate their emotions, the emotional experiences themselves may have differed in intensity. As past research has indicated that emotional intensity may impact on the success of certain cognitively demanding emotion regulation strategies [44, 45] this warrants further investigation.

## Conclusions

Current findings extend our knowledge of emotion regulation in BPF to provide an insight into what it is about emotion regulation attempts made by individuals with high levels of BPF that might make them less successful. It has been found, in this non-clinical population, that individuals with high levels of BPF utilise a range of emotion regulation strategies consistent with individuals reporting low levels of BPF. This suggests that although dysfunctional strategies may be used by individuals with high levels of BPF, their use is not specific to this population. Instead it appears that cognitions underlying the application of strategies is where these two groups differed, therefore it may be cognitions underlying strategy use rather that the strategies themselves that are problematic. It appears that, consistent with the process model of emotion regulation, there is a trend suggesting a lack of consideration for future unwanted emotion appears to result in these individuals regulating in a reactive rather than pro-active manners. This leads to regulation attempts during times of high emotional intensity, when ‘functional strategies’ are likely to be less effective [45]. In addition, differences in the desired function of emotion regulation attempts during times of high emotional intensity e.g. to *communicate internal negative experiences* may be problematic for interpersonal relationships. Finally, negative attention bias may make it more difficult for individuals to utilise some functional emotion regulation strategies, despite attempts to do so. These findings highlight factors that may contribute to the difficulties in emotion regulation associated with the presence of BPF. Following further investigation this information may be useful to inform support services both in the community and educational settings by highlighting potential targets for intervention in order to support individuals in learning to better manage their emotions. This might include supporting individuals to understand and recognise internal and external triggers for negative emotion, allowing early regulation; understanding why a negative attention bias may occur and develop skills in broadening attentional focus, and exploring alternative ways to communicate negative emotions. Supporting these individuals to understand and effectively manage their emotions may help to prevent emerging BPF in young adults from developing into stable and problematic personality features.

## Abbreviations

BA, bachelor of art; BPD, borderline personality disorder; BPF, borderline personality features; BSc, bachelor of science; DBT, dialectical behaviour therapy; PAI-BOR, personality assessment inventory – borderline scales; PANAS, positive and negative affect scale; PDQ-BS, personality diagnostic questionnaire – borderline scale.

## Footnotes

1 All participant numbers have been changed to preserve anonymity.
